# Swallowtail butterflies (Lepidoptera: Papilionidae) species diversity and distribution in Africa: The Papilionidae collection at the National Museums of Kenya, Nairobi, Kenya

**DOI:** 10.3897/BDJ.8.e50664

**Published:** 2020-04-02

**Authors:** Esther Kioko, Alex Mutinda Musyoki, Augustine Luanga, Mwinzi Duncan Kioko, Esther Wangui Mwangi, Lawrence Monda

**Affiliations:** 1 National Museums of Kenya, Nairobi, Kenya National Museums of Kenya Nairobi Kenya; 2 University of Nairobi, Nairobi, Kenya University of Nairobi Nairobi Kenya

**Keywords:** Swallowtail butterflies, Papilionidae, species diversity, distribution, Africa, National Museums of Kenya, Kenya

## Abstract

**Background:**

Species data from the Museum collections have been shown to be of great value as a tool for prioritising conservation actions in Africa (Fjeldsa and Tushabe 2005). The National Museums of Kenya (NMK) have an entomology collection, housed in 4,000 drawers in cabinets that contain over 1.5 million specimens, including the largest butterfly collection in Africa (Arnett et al. 1997). Lampe and Striebing (2005) demonstrated how to digitise large insect collections in order to make their associated label data into databases that can be used for functions, such as creating distribution maps. The NMK’s swallowtail butterflies' collection had not been digitised and thus there was a need to capture the label data to create a database that can be used for mapping the distribution of the species in Kenya and elsewhere. These data have addressed one of the most significant challenges to insect conservation i.e. the lack of baseline information concerning species diversity and distribution (Summerville and Crist 2003). These data have provided key historic papilionid species diversity and distribution data that can be used to monitor their populations, as butterflies are declining due to changes in land use, intensive agriculture and pestcide use, diseases and pest and climate change (Potts et al. 2016; Bongaarts 2019). The publication of the occurrence data records in GBIF has been undertaken, thus making the data available to a wider audience and promoting availability for use.

**New information:**

The swallowtail butterflies collection at the National Museums of Kenya was digitised from 2017–2019 and this paper presents details of the Papilionid collection at the Zoology Department, NMK, Nairobi, Kenya.

The collection holds 7,345 voucher specimens, consisting of three genera and 133 species. The collection covers the period between 1850 to 2019.

The distribution of the swallowtail butterflies, housed at the NMK, covers East Africa with 88%, Central Africa (6%), Western Africa (4%) and Southern Africa (2%).

## Introduction

Butterflies carry out essential ecosystem services which are necessary for human and environmental health. Butterflies are amongst the greatest number of flower visitors (Rader et al. 2015) and are important in the pollination of many leading cash crops globally. These pollinating insects have been undergoing a decline in abundance, occurrence and diversity in many parts of the world (Ollerton et al. 2014; Potts et al. 2016). According to Lawton et al. (1998), butterfly species richness in tropical forests decreases with anthropogenic disturbance. Butterflies are also key indicator species. According to Thomas (2005), butterflies can be used as indicator species since they are susceptible to their habitat patterns and fragmentation. Some butterfly species are disturbance-tolerant and can be found in areas altered by humans and are effectively tolerant to removal of the native vegetation (Davros et al. 2006). However, habitat-sensitive species have more specific requirements for habitat and vegetation composition to suit the needs of their other life stages and are often found only in relatively natural areas with native vegetation. In tropical forests, butterfly species richness has been shown to decrease with anthropogenic disturbance (Lawton et al. 1998). Heikkinen et al. (2009) showed that change in climatic parameters, such as increasing temperature, humidity and rainfall, could affect butterfly distribution. Though butterflies play important roles in the ecosystem in pollination and as indicator species, data for their diversity and abundance are limited. This project was undertaken to mine data from the NMK collection to make data available on the diversity and abundance of swallowtail butterfly species.

## General description

### Purpose

To create a freely accessible online resource for users.

## Project description

### Title

Assessment of Lepidoptera Pollinator Species Diversity Data in East Africa

### Personnel

Data mining from the National Museums of Kenya collection and additional field data from the Taita Hills ecosystem that forms the northernmost Eastern Arc Mountains was carried out by Esther N. Kioko, Alex M. Musyoki, Augustine Luanga, Duncan Mwinzi and others. Bioinformatics support for the data to be accessed online was provided by Esther W. Mwangi and Lawrence Monda.

### Funding

The project is supported by the JRS Biodiversity Foundation, USA

## Sampling methods

### Study extent

**Figure 1.**
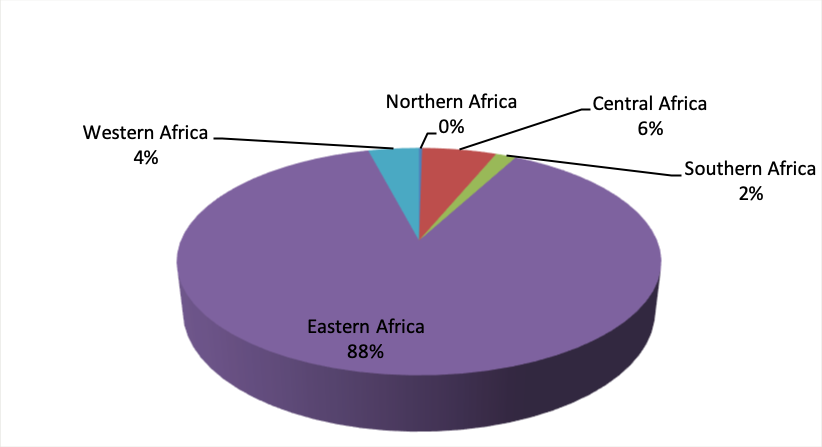
Papilionidae collection abundance per region.

The localities from which the Papilionidae specimens were collected are from all over Africa with East Africa leading with 88% as shown in Fig. 1.

### Sampling description

Papilionidae specimens housed at the NMK Invertebrate collection are as a result of multiple field expeditions and research projects. Most of the specimens lack information on the sampling protocol and, in case a certain method was used, then it was not indicated on the specimen label. The specimens were first catalogued and pinned; they were then preserved by drying in an oven.

### Quality control

Once a specimen was brought to the invertebrate collection, taxa experts revised the associated metadata i.e. species name (taxonomy) and locality. The geographical coordinates that were lacking, as is the case with old museum specimens, were obtained using a georeferencing webservice GEOLocate (Rios 2014). Verification of the taxonomic names was done by checking against the Butterflies of Kenya guide by Larsen (1996).

## Geographic coverage

### Description

The digitised swallowtail butterflies voucher specimens are all from Africa with East Africa at 88%, Central Africa (6%), Western Africa (4%) and Southern Africa (2%) as shown in Fig. 1.

## Taxonomic coverage

### Description

**Figure 2.**
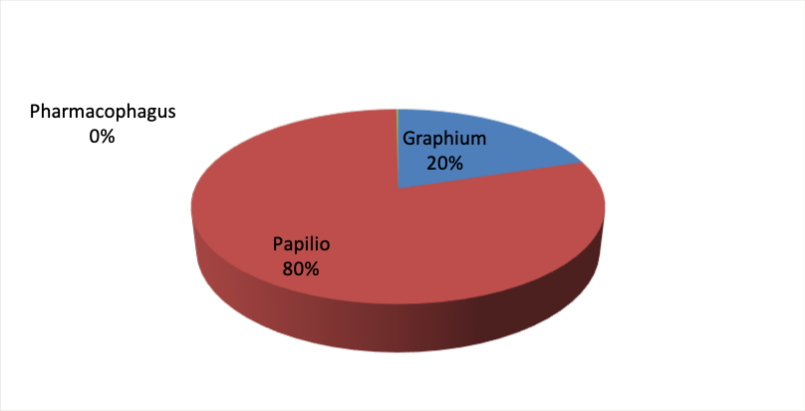
Papilionidae abundance per genus.

There are 7,345 Papilionidae voucher specimens that have been digitised and published in GBIF through the National Museums of Kenya's Integrated Publishing Toolkit (IPT) (Kioko et al. 2020). They belong to three tribes, namely Leptocircini, Papilionini and Troidini. The specimens belong to three genera: *Graphium, Papilio* and *Pharmacophagus* and consist of 133 species. The genus *Papilio*, belonging to tribe Papilionini, has 97 species out of 185 possible species represented in 5,847 specimens; this is followed distantly by the genus *Graphium*, belonging to the Leptocircini tribe, with 35 species out of a possible 80 species represented by 1,486 voucher specimens. The genus *Pharmacophagus*, belonging to tribe Troidini, has only one species represented by 12 voucher specimens (Fig. 2).

## Temporal coverage

### Notes

**Figure 3.**
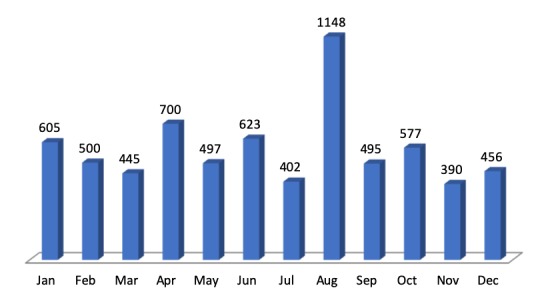
Papilionidae abundance at NMK collection monthwise.

The dates of the digitised papilionid collection ranged from April 1850 to May 2019. The voucher specimens were collected throughout the year with the highest collection numbers being in August with a record of 1,148 specimens, while the fewest being in November with 390 specimens, as shown in Fig. 3.

## Collection data

### Collection name

Invertebrate

### Collection identifier

https://www.gbif.org/grscicoll/collection/6c4bc956-850c-4f9e-b02f-9244a4376e30

### Specimen preservation method

Pinned

### Curatorial unit

Species collecting event

## Usage rights

### Use license

Creative Commons Public Domain Waiver (CC-Zero)

## Data resources

### Data package title

Lepidoptera

### Alternative identifiers

https://doi.org/10.15468/a1lgqs

### Number of data sets

1

### Data set 1.

#### Data set name

Occurrence data of swallowtail butterflies (Lepidoptera: Papilionidae) in the National Museums of Kenya Zoological collection in Nairobi

#### Data format

Excel

#### Number of columns

43

#### Download URL

https://www.gbif.org/dataset/3008196f-5ffc-496d-b11d-57f2124ee06b

#### Description

This resource is a digitised format of data on the occurrence of swallowtail butterfly species, housed in the Invertebrate Zoology Section, Zoology Department, National Museums of Kenya.

**Data set 1.** 

Column label	Column description
occurrenceID	An identifier for the Occurrence (as opposed to a particular digital record of the occurrence).
type	The nature or genre of the resource.
language	Language of the resource.
institutionCode	The name (or acronym) in use by the institution having custody of the object(s) or information referred to in the record.
collectionCode	The name, acronym, coden or initialism identifying the collection or dataset from which the record was derived.
basisOfRecord	The specific nature of the data record.
catalogNumber	An identifier (preferably unique) for the record within the dataset or collection.
individualCount	The number of individuals represented, present at the time of the Occurrence.
organismQuantity	A number or enumeration value for the quantity of organisms.
organismQuantityType	The type of quantification system used for the quantity of organisms.
year	The four-digit year in which the Event occurred, according to the Common Era Calendar.
month	The ordinal month in which the Event occurred.
day	The integer day of the month on which the Event occurred.
higherGeography	A list (concatenated and separated) of geographic names less specific than the information captured in the locality term.
continent	The name of the continent in which the Location occurs.
countryCode	The standard code for the country in which the Location occurs.
locality	The specific description of the place.
verbatimElevation	The original description of the elevation (altitude, usually above sea level) of the Location.
verbatimCoordinateSystem	The spatial coordinate system for the verbatimLatitude and verbatimLongitude or the verbatimCoordinates of the Location.
decimalLatitude	The geographic latitude (in decimal degrees, using the spatial reference system given in geodeticDatum) of the geographic centre of a Location.
decimalLongitude	The geographic longitude (in decimal degrees, using the spatial reference system given in geodeticDatum) of the geographic centre of a Location.
geodeticDatum	The ellipsoid, geodetic datum or spatial reference system (SRS) upon which the geographic coordinates given in decimalLatitude and decimalLongitude as based.
georeferencedBy	A list (concatenated and separated) of names of people, groups or organisations who determined the georeference (spatial representation) for the Location.
georeferencedDate	The date on which the Location was georeferenced.
scientificName	The full scientific name, with authorship and date information, if known.
higherClassification	A list (concatenated and separated) of taxa names terminating at the rank immediately superior to the taxon referenced in the taxon record.
kingdom	The full scientific name of the kingdom in which the taxon is classified.
phylum	The full scientific name of the phylum or division in which the taxon is classified.
class	The full scientific name of the class in which the taxon is classified.
order	The full scientific name of the order in which the taxon is classified.
family	The full scientific name of the family in which the taxon is classified.
genus	The full scientific name of the genus in which the taxon is classified.
specificEpithet	The name of the first or species epithet of the scientificName.
infraspecificEpithet	The name of the lowest or terminal infraspecific epithet of the scientificName, excluding any rank designation.
taxonRank	The taxonomic rank of the most specific name in the scientificName.
nomenclaturalCode	The nomenclatural code (or codes in the case of an ambiregnal name) under which the scientificName is constructed.
licence	A legal document giving official permission to do something with the resource.
modified	The most recent date-time on which the resource was changed.
references	A related resource that is referenced, cited or otherwise pointed to by the described resource.
institutionID	An identifier for the institution having custody of the object(s) or information referred to in the record.
recordedBy	A list (concatenated and separated) of names of people, groups or organisations responsible for recording the original Occurrence.
eventDate	The date-time or interval during which an Event occurred.
country	The name of the country or major administrative unit in which the Location occurs.

## Additional information

Africa is home to a rich biodiversity of butterflies that provide critical ecosystem services. However, most ecosystems in the continent are facing threats, including land use change, over-exploitation, environmental pollution, invasive alien species and climate change amongst others. These threats are leading to biodiversity loss and the need for data evidence to support decision-making on biodiversity conservation is critical. This paper has addressed the current limited capacity in publishing and using Digital Accessible Knowledge (DAK) to provide information for decisions on biodiversity conservation and sustainable use. The papilionid occurrence data forms a crucial baseline data that can be used for monitoring biodiversity trends and providing information about conservation decision-making processes.
